# Exploring intersectionality: an international yet individual issue

**DOI:** 10.1186/s13023-022-02255-3

**Published:** 2022-02-28

**Authors:** Zainab Alani

**Affiliations:** University of Glasgow Medical School, University of Glasgow, University Avenue, Glasgow, G12 8QQ Scotland

**Keywords:** Generalised Myasthenia Gravis, Rare disease, Intersectionality, Autoimmunity, Discrimination, Racism, BAME, Coronavirus

## Abstract

This article explores both reported and personal experiences of intersectionality within the healthcare system, which is often due to systemic inequalities as well as deep ingrained perceptions and opinions. With my perspective as both a medical student and rare disease patient battling generalised Myasthenia Gravis, I uncover and expose the aspects of intersectionality which are often brushed under the carpet. Moreover, I identify potential routes which we may collectively, as both clinicians and patients, embark upon to navigate our way out of this systemic snare. For those reading and engaging with this article, I endeavour to humanise the publicised figures surrounding rare disease and emphasise that within each figure there are patients, just like myself, who too may be experiencing the multifactorial issues arising from intersectionality. Furthermore, the coronavirus pandemic has highlighted and emphasised the pre-existing divide in the treatment of societal groups, for those both receiving and delivering care. We have long attempted to plaster over this chronic wound however the distressing outcomes of this pandemic have forced us to address this shameful truth from its core. Intersectionality is a disease which is destroying our healthcare system from within. However, unlike many rare diseases, intersectionality can be abolished.

## Intersectionality exposed

It is one matter to explain intersectionality, and another altogether to experience it. In 2015, the Oxford English Dictionary defined intersectionality as *“The interconnected nature of social categorizations such as race, class, and gender, regarded as creating overlapping and interdependent systems of discrimination or disadvantage”* [1]. As powerful and impactful as this literal definition may be, it pales into insignificance when compared with the breath-taking blow felt when enduring intersectionality. As a patient living with a rare disease which only affects 15 in every 100,000 people [2], namely generalised Myasthenia Gravis (MG), I myself faced intersectionality when enduring the physically and mentally draining process of diagnosis where I was left questioning both my able yet anxious self and aspects of our very own extraordinary yet flawed healthcare system. But how can we fundamentally act upon such flaws when those facing these issues do not feel empowered enough to voice it?

## The distinguishing differences

In order to understand the disadvantages that victims of intersectionality face, we have to understand the differences that make us, and I say “us” as I regrettably know from experience, stand out. Race, religion, and gender are often the forefront themes when it comes to uncovering issues regarding discrimination, however, many more potent factors are often brushed under the carpet such as income and age. In a 2017 article, the impact of income was highlighted and the dichotomy outlined here between races is, in my opinion, utterly distressing; higher earnings in white populations were associated with better care yet in black communities, despite striving for and attaining higher earnings to attempt to bridge this overwhelming gap between white and black communities, this achievement was rewarded with lower care levels [3]. An example of the latter less discussed factor, age, is a more personal matter. When going through the process of diagnosis at the age of 15, I was suffering from extreme fatigue secondary to my MG however I was dismissed, by more than one healthcare professional, as being a “teenager” or “being lazy”. This, understandably, left me feeling defeated, embarrassed, and ashamed; how dare I be wasting valuable NHS resources with my simple fatigue? Had it not been for the support of family and other healthcare professionals, my diagnosis would have gone unnoticed and would have been overlooked until it would have been too late for preventive action. Such toxic associations—whether linked with age, race, religion, or any other factor—feed into creating a discriminatory environment which pushes those from minority backgrounds towards the periphery. Ultimately, this has resulted in healthcare inequalities between ethnic minority and white communities which has been painfully highlighted during the COVID-19 pandemic [4].

## Coronavirus as a catalyst for inequality

Disconcertingly, the impact of the pandemic has compounded the issue of intersectionality within our chronically ailing healthcare system. Although immediate and overt damage has been done in regards with the harrowingly increased loss of life from black and minority ethnic (BAME) groups [5, 6], we must collectively attempt to make a concerted effort to minimise the inevitable distrust that the pandemic has left in its wake. This painful experience has shone light on the shortcomings of the NHS in regards with providing adequate protection for those belonging to BAME groups and, understandably, those belonging to such communities—whether patients or doctors—will be plagued with feelings of abandonment and disillusionment with the health and social care system [7, 8]. The underlying issues of intersectionality, combined with the negative experiences of the pandemic, put such groups at a colossal disadvantage when it comes to seeking care. Intrinsically, they may not want to approach the NHS again for fear of a repeated let down, mirroring the experience of the pandemic. Worse still, this can lead to community-wide avoidance of seeking support which would worsen and deepen the healthcare divide and disparities which already exist. To remedy this, the NHS must combat and solve these highlighted issues by actively engaging with these groups—whether through community campaigns, targeted support, or nationwide education programmes—to inform the public about the advancements in equality of care. It is our duty to ensure that such communities do not lose the amazing healthcare that is available at their fingertips which they deserve just as equally as any other citizen.

## Discrepancies in amenities, access, and life expectancy

Nonetheless, it cannot be denied that these problems existed beforehand and that the pandemic merely amplified rather than initiated such a discriminatory divide within the healthcare system. Ashamedly, several studies in Europe have confirmed this unsettling link between discriminatory behaviour and lesser standards of care [9]. Particularly in the UK, we are guilty of not tackling the disparity in life expectancy, between wealthy and poorer areas, vigorously enough. This form of discrimination is often overlooked and rather than being associated with less resources being employed at the point of care, this discrepancy arises due to poorer communities not knowing how or simply not being able to access preventive care. A study in Scotland found that not only does this life expectancy gap differ between rich and poor, but a gap also exists amongst men and women within each area respectively; in affluent areas, women and men have 22.6 and 23.8 more years of health, respectively, compared to those living in areas of deprivation [10]. Evidently, if such populations are unable to access preventive care services such as screening programmes, lifestyle advice or community improvement projects, then this will undoubtedly result in decreased health and, with time, onset of disease. The wide-reaching impacts of intersectionality are tiring and draining and are, quite literally, ageing certain groups more rapidly than others. However, we must revitalise these populations through renewed hope of a better future; we must signpost preventive care services better, we must ensure better outreach to such communities and, foremost, we must bridge this life expectancy gap.

## Intersectionality and I

Despite having addressed some of these issues arising from intersectionality in their own right, it must be understood that these problems rarely occur in isolation but rather, are compounded into a single patient experience. Now, as this discussion draws to a close, I would like to reflect on my own experience as a rare disease patient who has been subjected to several of the traps spun in the web of intersectionality. As a young, female, Arab, Myasthenia Gravis patient I have experienced various forms of discrimination. As aforementioned, as a young patient my concerns were taken lightly and automatically deemed secondary to the misinformed stereotype of the lazy, carefree, lifestyle of a teenager. Yet, at the same time, I was assumed to be an adult in respect with no longer needing the support of family and friends, exemplified when I was delivered a lifelong diagnosis over the phone alone at the age of 15. As a female, it was also assumed that my fatigue and myasthenic symptoms were merely due to ‘time of the month’. And finally, as an Arab, I have faced the most prominent forms of discrimination, possibly surprisingly in the form of racial micro-aggression; whether manifesting as healthcare professionals not attempting to pronounce my name properly during appointments or as simple acts while attempting to create natural conversation such as asking, “*So where are you really from?”* Interestingly, in stark contrast to the vast majority of rare diseases which are genetic or Mendelian in origin, Myasthenia Gravis is an autoimmune condition whereby the body mistakenly produces autoantibodies against the acetylcholine receptor (AChR) on the motor end plate of muscle fibres, resulting in fatigable muscle weakness [11]. MG is one of the 20% of rare diseases which are non-genetic in origin [12]; undoubtedly, this too contributed towards littering my diagnosis journey with yet more hurdles and obstacles to overcome. Moreover, although not vastly dissimilar, reaching a diagnosis for autoimmune diseases takes roughly 4.5 years which is marginally longer than the respective timeframe for rare diseases in general which is roughly 4 years [13–15]. Nevertheless, for both genetic and non-genetic rare disease patients constituting the 1 in 17 affected by rare diseases during their lifetime, the aforementioned difficulties which we must tackle are universal [16, 17].

## Inherent intersectionality issues within the wider rare disease community

Unfortunately, yet unsurprisingly, many parallels can be drawn between my own experiences and those of my fellow rare disease patients as exemplified by a study showing that ultra-rare diseases, particularly those in children, are not provided adequate funding and resources for research purposes [18]. This, once again, highlights how age can be a prominent factor contributing to intersectionality. To contextualise how serious this issue is, 75% of rare diseases impact children and, of those affected, 30% will not live beyond the age of 5 [19]. Furthermore, regarding the subject of race, a study involving sickle cell patients and their families found that a staggering 50% believed that their race and ethnicity had a significant impact on the quality of care they received [20]. Further catalysing this issue of inequalities in care is the problem whereby accessing appropriate treatment often proves arduous, which is coupled with the difficulty that many healthcare professionals often refuse to administer treatment in the first instance [21]. Disconcertingly, what is of even more concern is that the 2021 Rare Barometer Survey outlined that an overwhelming 39% of respondents felt discriminated against solely on the basis of their rare disease [22]. All of these perpetual and systemic discrimination factors, along with the often unchanging rare disease diagnosis, compound to create a negative mental environment whereby the patient is plunged into the depths of feelings of worthlessness, sadness and powerlessness. Therefore, in order to tackle intersectionality, we must not only take down each brick of discrimination that blocks the path to equality but bring down the whole wall simultaneously.

## Advancing and innovating rare disease care

Moving forward, it cannot be denied that there is more that could be done to prevent intersectionality from compounding the countless issues that rare disease communities already face. With the European Union showing solidarity with the rare disease community by investing almost €2 billion in over 300 projects during the last 15 years, we are undoubtedly on the path to curing many of these enigmatic conditions [23]. However, the actions taken to combat the chronic and seemingly incurable issue of intersectionality do not have to be as monumental. For example, this could be as simple as healthcare professionals regularly asking patients and carers for their opinions on the care they are receiving. This could be seamlessly incorporated into check-up and follow-up appointments as part of routine care to better identify issues before they become fully established, elevating patient experiences. Moreover, more open and honest conversation must be facilitated between patients and healthcare providers to allow potential issues to be discussed, however, this is only possible if we collectively tackle the stigma associated with discussing racism and discrimination. Those who perpetuate racist and discriminatory behaviour, which feeds into creating a culture of isolating intersectionality, often fail or refuse to recognise that they are in the wrong. Sadly, this is often due to the blame culture present within our society today. Now, we must move away from such trapping behaviours and adapt to become proactive; we must learn from our mistakes, we must learn to respect everyone and, above all, we must learn about each other.

## An international and individual issue, yet to be addressed

Striving for healthcare excellence means that we must address the divisions and discrimination, often caused by systemic intersectionality issues, which are deeply ingrained within current ‘accepted’ standards of practice. As highlighted earlier, intersectionality is not only an international issue hindering our generation but is also an individual issue for myself as a rare disease patient. As both a Myasthenia Gravis patient and medical student, and aspiring future doctor, I hope for the wider global population of rare disease patients and myself that we—healthcare professionals and patients in unison—can collectively bridge the gap that intersectionality has generated in our healthcare system and eradicate the international, yet individual, issues arising from intersectionality.

## Data Availability

See References.

## Abbreviations

MG: Myasthenia Gravis
NHS: National Health Service
COVID-19: Coronavirus Disease 2019
BAME: Black and Minority Ethnic
